# The role of cancer and immunosuppression in pneumocystis pneumonia: a large comprehensive population-based study

**DOI:** 10.1007/s15010-026-02802-1

**Published:** 2026-05-06

**Authors:** Fabian Reichel, Falko Tesch, Saskia Berger, Martin Seifert, Dirk Koschel, Jochen Schmitt, Martin Kolditz

**Affiliations:** 1 Medical Department I, Division of Pneumology, University Hospital Carl Gustav Carus, TUD Dresden University of Technology, Fetscherstr. 74, 01307 Dresden, Germany; 2 East German Lung Center / Ostdeutsches Lungenzentrum Dresden-Coswig, Dresden, Germany; 3 Center for Evidence-Based Healthcare, Faculty of Medicine, University Hospital Carl Gustav Carus at TUD Dresden University of Technology, Dresden, Germany; 4 Hospital Pharmacy, University Hospital Carl Gustav Carus, TUD Dresden University of Technology, Dresden, Germany; 5 Department of Internal Medicine and Pneumology, Lungenzentrum Coswig, Germany

**Keywords:** Pneumocystis, PCP, PJP, Pneumonia, Immunosuppression, Steroids, Mortality

## Abstract

Although pneumocystis jirovecii pneumonia (PJP) in non-HIV patients is increasingly recognized, comprehensive data on immunosuppressive drivers remain limited. We conducted a population-based cohort study using a German health insurance’s claims data (2015–2023). PJP, comorbidities, immunocompromising conditions and therapies were identified by means of ICD-10-GM-, ATC- and OPS-coded records. 922,200 individuals were assessed for PJP occurrence, hospitalization and 30-day all-cause mortality. An Andersen-Gill model adjusted for key covariates. We identified 171 PJP episodes, with hospitalization in 88.3% and mortality in 24.5%. Frequent immunocompromising causes were hematologic cancers (42.6%), immunosuppressants (28.7%) and steroids (28.7%). Immunosuppression rather than typical CAP risk factors strongly predicted all outcomes (HR 49.9 [31.5–78.9]). Robust associations with PJP occurred for HIV (HR 67.5 [19.4–235]), systemic steroids (> 20 mg prednisone daily equivalent (HR 26.2 [13.2–52.1])) and hematologic cancers (HR 8.5 [4.5–16.3]). Mortality was driven by hematologic cancers (HR 13.9 [4.8–40.2]) and high-dose steroids (HR 8.6 [1.6–47.1]). Malignancy-related immunosuppression and steroids critically affect PJP risk and prognosis.

## Introduction

Pneumocystis jirovecii pneumonia (PJP), an opportunistic lung infection, is generally recognized as an AIDS-defining disease in HIV patients. As incidence and mortality in patients suffering from HIV has consistently decreased in the past, it has become increasingly more prevalent in otherwise immunocompromised individuals [1–5]. Thus, detailed data on development and prognosis of PJP and specific risk factors associated with different conditions of immunosuppression such as cancer or -related therapy is urgently called for.

## Methods

The following report is a subgroup analysis of our previously published retrospective population-based cohort study on risk factors for CAP and its outcome with an extended observation period for PJP and particular consideration of malignancies and immunosuppression. Detailed methodology including the definitions of different causes of immunosuppression can be obtained from thereabouts [6]. This investigation was approved by the TUD Dresden University of Technology ethics committee (EK143052018). German health insurance’s claims data from 2015 to 2023 was pseudonymously investigated. PJP was defined as hospitalization and an ICD-10-GM-coded main discharge diagnosis of B59 (2015–2018) or B48.5 (2019–2023), a main discharge diagnosis of sepsis (A40.x-A41.x) combined with a secondary diagnosis of B59 or B48.5, or an ambulatory diagnosis of B59 or B48.5 followed by a prescription of a PJP-effective anti-infective drug (ATC J01EE01, J01EA01/J04BA02, J01FF01/P01BA03, P01BB51, P01CX01). Mortality was defined as death of any cause within 30 days of PJP diagnosis. The analysis was adjusted for age, sex, long-term care, type of community, pneumococcal/influenza vaccination, chronic comorbidities and immunocompromising factors. Immunosuppression could be obtained by both an underlying condition (hematologic malignancies, stem cell or solid organ transplantation, neutropenia, HIV or primary or humoral immune deficiency syndromes) and/or a drug-induced deficiency (prescribed antineoplastic drugs, immunosuppressants or systemic steroids using a prednisone daily dose equivalent (PDDE) > 10 mg) as documented by corresponding ICD-10-GM, ATC and OPS codes. The Andersen-Gill model was used to calculate estimates displayed as hazard ratios (HR) and their 95%-confidence intervals (CI). The analyses were performed with R (v3.6.3) and RStudio (v2023.09.1 + 494) using the survival and forestplot package.

## Results

We acquired a total study population of 922,200 individuals. Of these, 157 had coded PJP during the study period. Excluding 5 patients with > 4 (9–26) coded PJP episodes due to presumably incorrect diagnoses, 152 patients with 171 PJP episodes were evaluated (Table 1). The hospitalization rate was 88.3% (*n* = 151). All-cause 30-day mortality was 24.5% (*n* = 42). Among immunosuppressive conditions in PJP episodes, hematologic malignancies were most frequent (42.6%) followed by immunosuppressant therapy (28.7%) and systemic steroids with a PDDE ≥ 10 mg (28.7%). HIV infection was present in 13.9% of immunocompromised PJP-episodes.

**Table 1:** Characteristics of the PJP and control episodes

Total episodes	PJP episodes	Relative share [%]	Control episodes	Relative share [%]
	171	100	5,756,659	100
Male	100	58.5	2,394,598	41.6
Female	71	41.5	3,362,061	58.4
Year of birth ≥1950	87	50.8	2,500,145	43.4
Year of birth < 1950	84	49.1	3,256,514	56.6
Long-term care required	65	38.0	1,509,824	26.2
City (compared to provincial/rural)	71	41.5	1,627,274	28.3
Cardiovascular disease	76	44.4	2,195,131	38.1
Neurological disorder	43	25.2	1,820,787	31.6
Pulmonary disease	64	37.4	1,054,993	18.3
Renal disease	60	35.1	1,232,665	21.4
Gastrointestinal disease	41	24.0	936,382	16.3
Diabetes mellitus	56	32.8	2,168,923	37.6
Rheumatic disease	44	25.7	545,062	9.5
Solid tumor w/o immunosuppression	25	14.6	706,784	12.3
Immunosuppression in general	108	63.3	410,680	7.1
Immunosuppressive condition	82	48.0	217,137	3.8
Active hematologic malignancy	46	26.9	133,250	2.3
Solid organ transplantation	19	11.1	29,899	0.5
Stem cell transplantation	8	4.7	4,402	0.1
Neutropenia	7	4.1	35,733	0.6
HIV-infection	15	8.8	4,564	0.1
Primary and humoral immune deficiency	16	9.4	31,090	0.5
Antineoplastic drugs	16	9.4	45,824	0.8
Immunosuppressants	31	18.1	136,459	2.4
Systemic steroids PDDE 10–20 mg	6	3.5	42,696	0.7
Systemic steroids PDDE > 20 mg	25	14.6	39,984	0.7

Patients’ coded medical timelines were split into a new episode each time an additional diagnosis emerged, an obsolete diagnosis was removed or a relevant change in medication occurred during the observation period

**Fig. 1:**
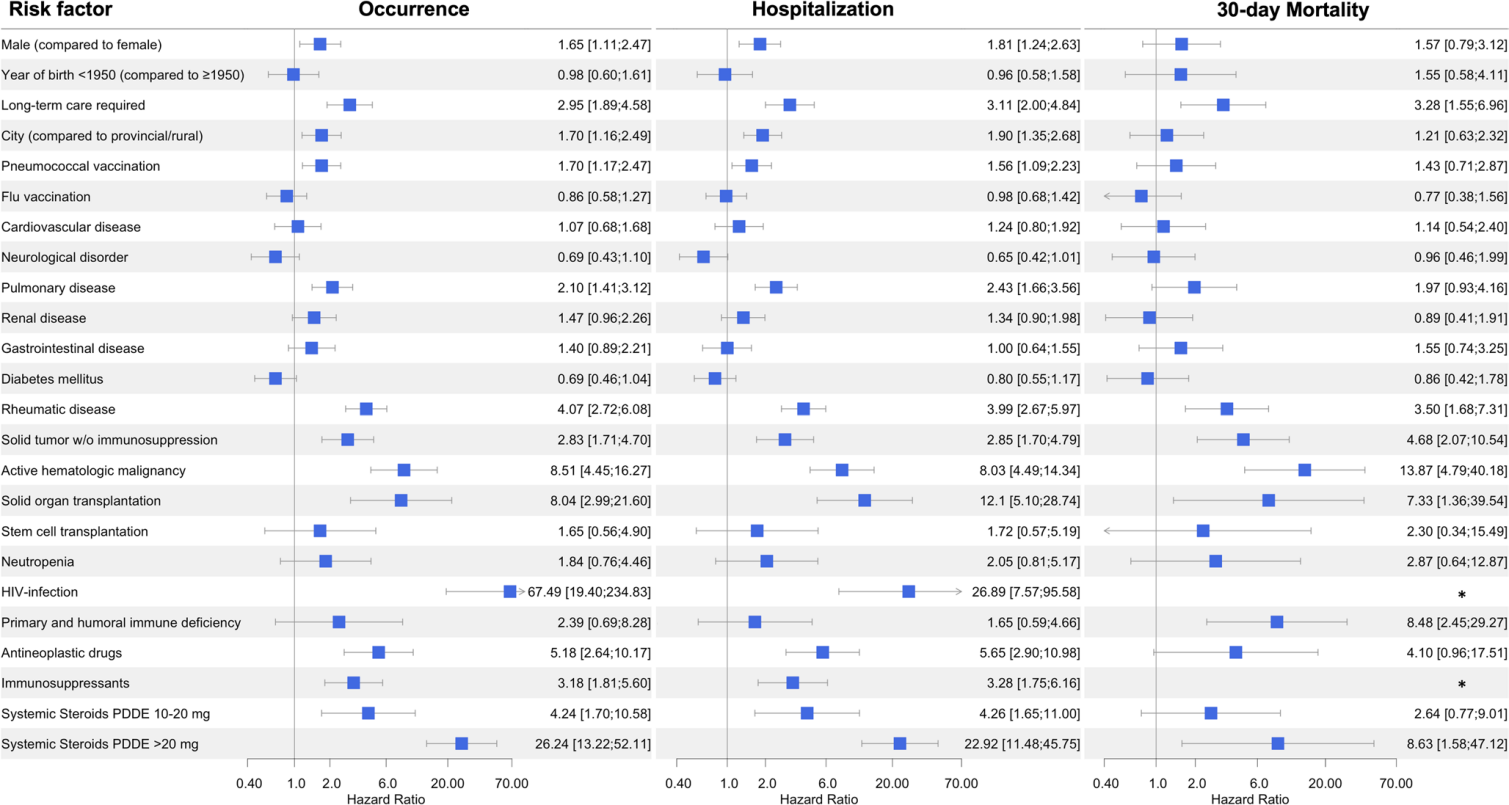
Risk factor evaluation for occurrence of PJP, required hospitalization and all-cause 30-day mortality. 171 PJPs out of ∼ 5.8 Mio. total recorded observation episodes were analyzed. Hazard ratios (HR) and corresponding 95% confidence intervals were calculated using the Andersen-Gill model and are displayed as forest plot. PDDE – prednisone daily dose equivalent. * – no deaths among patients under immunosuppressants or with HIV were detected

Among comorbidities, patients suffering from rheumatic diseases were most vulnerable to contract PJP (HR 4.01) while solid malignancies displayed the highest risk for a fatal outcome (HR 4.68). Apart from pulmonary diseases (HR 2.10), other organ disorders were not principally associated with PJP (Fig. 1). However, all above-named risks were dominated by an underlying immunosuppression. Overall, immunocompromised individuals were at risk with a HR 60.46 [95%-CI 37.52–97.43] and displayed significantly poorer prognosis resulting in hospitalization (HR 49.87 [95%-CI 31.52–78.91]) and/or death (HR 54.84 [95%-CI 22.92–131.20]). Amidst specific immunosuppressing factors, HIV infection entailed by far the greatest risk for PJP (HR 67.49) followed by systemic steroids using a PDDE > 20 mg (HR 26.24), hematologic malignancies (HR 8.51), solid organ transplantation (HR 8.04) and antineoplastic treatment (HR 5.18). Hospitalization was particularly required in HIV infected individuals (HR 26.89) as well as patients on high-dose systemic steroids (HR 22.91) or after solid organ transplantation (HR 12.10). All-cause 30-day mortality was robustly associated with hematologic malignancies (HR 13.87) and systemic steroids (PDDE > 20 mg; HR 8.63).

## Discussion

This study analyzed a substantially large cohort for PJP and its risk factors for occurrence and outcome including a granular investigation of immunosuppression e.g. due to malignancies.

Analyzing a health insurance’s coded medical information gave us the capability to access an extensive, standardized database providing a sufficient sample size for a comprehensive risk evaluation of a rare disease while minimizing selection or recall bias. Yet, a major limitation is the lack of actual medical reports and PJP validation by microbiological and other diagnostic findings. This issue was partially approached by requiring targeted treatment prescriptions for PJP following an outpatient diagnosis.

Although considered a rare disease, hospitalization and mortality of PJP remain remarkably high at 88.3% and 24.5%, respectively, matching mortality data from a German multi-center study [1]. As recently published [7], we confirmed that typical risk factors for CAP such as age and long-term care are only of subordinate importance for contracting PJP. In contrast, immunosuppression emerges as the major determinant for an increased risk of PJP. Although historically of particular significance as an AIDS-defining disease PJP incidence has declined in the past years in individuals with HIV. In accordance with the earlier observed lower mortality [1–3], we did not detect any deaths within 30 days of PJP diagnosis in HIV-positive individuals. Nevertheless, HIV constitutes a substantial threat, demonstrating the highest risk of PJP occurrence and subsequent hospitalization in our cohort.

Over the past decades, the population of patients with non-HIV-related immunosuppression has grown substantially, reflecting improved survival of immunocompromising diseases and the expanding application of immunosuppressive therapies. Consequently, PJP rates in non-HIV patients have increased [1]. However, the type and degree of immunosuppression substantially affect risk and outcome of PJP. Hematologic malignancies and solid tumors, organ transplantation, rheumatic as well as lung diseases and therapies using antineoplastics, immunosuppressants and systemic steroids were associated with occurrence of PJP. Yet prognosis was mainly determined by underlying hematologic or rheumatic diseases, solid organ transplantation, immune deficiencies and systemic steroids while as in HIV patients no deaths occurred in patients receiving immunosuppressants. Along with published data suggesting a particularly high PJP susceptibility under steroid therapy, we detected a dose-dependent association [7, 8]. In fact, using a PDDE of > 20 mg was identified as the strongest determinant for PJP among pharmaceutics and furthermore had the most severe effect on mortality. It may therefore reasonably serve as threshold for additional PJP prophylaxis. Our data mirrors previous results showing that patients with rheumatic or pulmonary diseases and malignancies, often combined with steroid treatment, present with severe courses and poor outcomes attributable to PJP [1, 3, 5, 7]. While hematologic malignancies remain a major risk for PJP and poor outcome, a lower rate of PJP in these patients has been previously reported [1, 2]. It is noteworthy that guidelines in hematology, stem cell or organ transplantation routinely address PJP and its prophylaxis whereas guidelines on rheumatic or chronic lung diseases and malignancies have thus far failed to address this [3, 7, 9].

In conclusion, our large population-based subset study adds comprehensive knowledge to the growing body of evidence on risk factors for PJP, highlighting the impact of specific comorbidities and immunosuppressive conditions for disease occurrence and prognosis. These results support both a more targeted diagnostic workup and prophylactic strategies for patients at risk.

## Data Availability

Datasets and materials other than presented in the manuscript are not publicly available due to general and individual privacy interests.

## Declarations

### Artificial intelligence involvement

No artificial intelligence was used for the creation of this article.
